# Microbiome Changes in Children Treated under General Anesthesia for Severe Early Childhood Caries: Pilot Study

**DOI:** 10.3390/children10010030

**Published:** 2022-12-24

**Authors:** Tal Ratson, Nurit Dagon, Sigalit Blumer, Nir Sterer

**Affiliations:** 1 Department of Paediatric Dentistry, Maurice and Gabriela Goldschleger School of Dental Medicine, Sackler Faculty of Medicine, Tel Aviv University, Tel Aviv 6997801, Israel; 2 Department of Prosthodontics, Maurice and Gabriela Goldschleger School of Dental Medicine, Sackler Faculty of Medicine, Tel Aviv University, Tel Aviv 6997801, Israel

**Keywords:** severe early childhood caries, general anesthesia, microbiome

## Abstract

A full-mouth radical dental treatment under general anesthesia is a common approach for treating severe early childhood caries (S-ECC). However, previous study showed recurrence of the disease in 80% of cases within 12 months. The aim of the present study was to examine the changes in microbial composition of the dental biofilm of these children following treatment. Dental biofilm samples from five children (mean age 45.4 ± 10.1 months) were taken before and three months after treatment and analyzed for microbial composition using Next Generation Sequencing of the microbial DNA extracted from these samples. Although some reductions in the abundance of caries-pathogenic bacteria (e.g., *Streptococcus mutans*, *Streptococcus sobrinus*, *Rothia dentocariosa* and *Scardovia wiggisiae*) were seen in the post-treatment follow up samples, these reductions were for the most part not statistically significant, and these bacteria remained well above detection levels. Taken together, the results of the present pilot study suggest that the dental treatment alone is not enough to reduce the caries risk status of these children and that a more comprehensive approach should be considered.

## 1. Introduction

The oral microbiome is an intricate assortment of microbial species, the composition of which affects the advancement of oral diseases and health [1]. The oral cavity, which harbors more than 800 different bacterial species, is one of the most diverse and important habitats of the human microbiota [2]. The oral cavity of a healthy person harbors a relatively stable microbial community that maintain symbiotic or eubiotic relationship with the host, but when this balance is disturbed, it may result in pathogenic conditions such as caries and periodontal diseases [3,4]. Although the oral microbiome constitutes a natural line of defense together with saliva and other immunological factors, it has limited capability to recover from a disease state that results from over-abundance of specific oral pathogens [1].

Dental caries is one of the most common chronic diseases in children around the world. Therefore, there is a need for gaining a better understanding of how oral microbial communities may affect children’s oral health status. Early childhood caries (ECC) and its severe form (S-ECC) were first termed in the 1990s [5]. ECC is defined by the American Academy of Paediatric Dentistry (AAPD) as the presence of dental caries affected teeth (decayed, missing or filled) in the primary dentition of children under the age of six. In contract, S-ECC, its severe form was characterized by the presence of smooth surface lesions under the age of three or involvement of multiple sites including maxillary anterior teeth under the age of five [6]. A national survey conducted in 2012 showed that ECC was highly prevalent in preschool children from lower socioeconomically populations and had a strong impact on the child’s development and well-being resulting in emergency treatments, loss of school days and reduced quality of life [7,8]. ECC has considerable immediate and future implications. In addition, caries severity in primary teeth may be a predictor for caries experience in the permanent dentition [9].

Despite the emergence of minimally invasive treatment approaches to caries management using means such as silver diamine fluoride (SDF) [10], the treatment of ECC still poses a challenge. Young children are often unable to cooperate in an office setting, and are therefore at a risk for caries progression to pain and disseminated odontogenic infection. When a child is unable to cooperate with a standard dental treatment and caries burden is substantial, general anesthesia (GA) might be warranted [11]. However, treatment under general anesthesia (GA) for extensive dental repair/restoration is time consuming and costly. Furthermore, the experience can have a traumatic effect on the child and its family [12].

There is ample evidence linking the bacterial species *Mutans streptococci* with dental caries in humans, and, together with Lactobacilli, they are regarded as odontopathogenes [13]. A recent systematic review conducted on 87 studies with a sum total of over a million cases reported on risk factors associated with ECC including socioeconomic, dietary and oral hygiene factors concluded that high levels of *Streptococcus mutans* were among the strongest risk factors found [14]. Furthermore, another study on microbiome associated with ECC has shown that *Streptococcus mutans* was the most discriminatory bacterium between health and disease status of ECC [15]. The number of active carious sites, such as lesions, fillings and other retention sites in the oral cavity, are thought to increase microbial colonization [16,17,18]. Therefore, it is assumed that extensive dental treatment including the extraction and restorative elimination of cavities and retentive sites should diminish the number of cariogenic microorganisms [19].

A full-mouth comprehensive dental treatment under GA is an effective method for treating multiple sites affected by dental caries in children suffering from S-ECC. However, previous study reported that 79.9% of children treated for S-ECC under GA showed reoccurrence of the disease within 12 months [20]. There is not a lot of information about the changes in microbiome of children after comprehensive dental treatment under GA and the mechanism by which these changes influence the caries status of a population with S-ECC.

Thus, the aim of the present study was to investigate the effects of comprehensive dental rehabilitation under GA on the microbiome of children with S-ECC and to determine whether this approach is efficient in reducing the risk for caries recurrence.

## 2. Materials and Methods

### 2.1. Study Population

Five children between the ages of three and six years old were included in the study. All of them were healthy children diagnosed with S-ECC based on a preliminary clinical and radiographical examination and scheduled to undergo dental treatment under GA. Treatment took place in the department of pediatric dentistry, School of Dental Medicine, Tel Aviv University. All caries affected teeth were treated using composite-resin restorations, pedoform crowns, SCC, pulpotomies, pulpectomies or extractions. Dental plaque removal and a fluoride varnish application (5% NaF) were implemented in all cases. Following treatment parents received oral and written instruction regarding the treatment and follow-up appointments, including dietary and oral hygiene instructions. Children that had taken antibiotic a month prior to the study, and children that did not arrive to the follow-up 3 months after the GA were excluded from the study.

### 2.2. Experimental Protocol

The study protocol was approved by the Tel Aviv University Institutional Ethics Committees (#0001771-1, 9 July 2020), and informed consent was attained from the parents of all participating children. Study was conducted in accordance with the CONSORT statement.

General information, such as age, gender, health status and dmfT (WHO criteria) as well as dietary and oral hygiene habits, was obtained.

Dental plaque samples were taken at baseline, and at 3 months after comprehensive restorative and extraction therapy under GA. Plaque samples were taken from the *buccal* and interproximal areas of the primary *Maxillary* molars with a sterilized curette [21], placed in 400 μL PBS (Phosphate-buffered saline), and frozen at −20 °C until microbial analysis.

### 2.3. Deoxyribonucleic Acids (DNA) Extraction

DNA was extracted from samples using 400 μL GT lysis buffer and 20 μL proteinase K (Magcore Genomic DNA Tissue Kit, Taipei, Taiwan) in beadbeating tubes type C (Geneaid, Taipei, Taiwan). Beadbeating was performed for 2 min using a Biospec machine. Samples were then incubated at 60 degrees for 2 h, and extracted on Magcore machine (RBC Bioscience, Taipei, Taiwan) using Magcore Genomic DNA Tissue Kit cartridges and protocol.

### 2.4. Polymerase Chain Reaction (PCR) Amplification and Sequencing

DNA was quantified by nanodrop and 3 μL (~15 ng) was used as template for initial PCR. Amplification was performed using Kapa Hifi Hot start ready mix using custom primers covering the V4 region primers from Earth Microbiome Project containing CS1/CS2 adaptors for 20 cycles in a volume of 15 μL. Bacterial 16 s rDNA were amplified using the 16 s forward primer 515 F (CS1-5′-ACACTGACGACATGGTTCTACANNNNCCTACGGGAGGCAGCAG) and reverse primer 806 R (CS2-5′-TACGGTAGCAGAGACTTGGTCTGGACTACHVGGGTWTCTAAT).

A 2 μL sample from PCR1 amplified sample containing CS1/CS2 adaptors was amplified for 10 cycles in 10 μL using Fluidigm Access Array Barcode library according to manufacturer’s protocol (2 μL barcode per reaction). DNA was purified using Kapa Pure Beads at a ratio of 0.8× and quantified with qubit using Denovix DsDNA high sensitivity assay (Wilmington, DW, USA). DNA size and integrity was quantified by Tapestation using Agilent DNA screen tape and reagents.

### 2.5. Sequencing Data Analysis

Samples were run on a dedicated Miseq (Illumina, San Diego, CA, USA) machine [22] with 30% PhiX using MiSeq Reagent Kit v2 500PE. Demultiplexing was performed using bcl2fastq with default parameters allowing for 1 mismatch. Data were then mapped to PhiX using bwa and unmapped reads were quantified, collected and examined using fastQC.

Demultiplexed reads were uploaded into CLC genomics workbench (Quigen, Hilden, Germany), and analyzed using their 16S microbiome pipeline. The analysis workflow consisting of quality filtration of the sequence data, and operational taxonomic unit (OTU) clustering was performed with default parameter settings. The adaptor sequence was removed, and the reads with a quality score lower than 25 or length < 150 were discarded. The maximum number of acceptable ambiguous nucleotides was set to 2, and the length of the reads was fixed at 200–500 bp. Chimeric sequences and singletons were detected and discarded. The remaining unique reads were used for OTU clustering, which was performed by alignment to the SILVA database at 97% sequence similarity.

### 2.6. Statistical Analysis

The data were described at the phylum, genus and species level in terms of abundance and prevalence. The differences between pre-treatment and post-treatment samples were analyzed using Kruskal–Wallis test with the pairwise Wilcoxon test among subclasses (PAST statistics, Oslo University, Norway). *p* value < 0.05 was considered statistically significant.

## 3. Results

Patients’ information is summarized in Table 1. A total of five children (mean age 45.4 ± 10.1 months, one female) completed the study. All the children were diagnosed with S-ECC with no prior dental treatment and average Total dmfT of 13.4 ± 2.7. All of the decayed teeth were treated, the majority of them were restored (85%) and the rest were extracted. In total, dental treatments included 13 composite resin restorations, 29 stainless still crowns, 16 pedoforms, 10 pulpotomies, 3 pulpectomies and 10 extractions.

For both pre- and post-treatment plaque samples tested, all the bacterial species identified could be classified into seven phyla, 36 genera and 89 species. Four phyla; *Firmicutes*, *Proteobacteria*, *Actinobacteria* and *Bacteriodetes* were found in all of the samples and accounted for about 90% of the sequences. The phylum *Firmicutes* accounted for more than a third of bacterial species found in the samples. Four genera; *Streptococcus*, *Veillonella*, *Actinomyces* and *Rothia* were found in all of the samples, out of which *streptococcus* and *Actinomyces* were the most abundant ones.

**Figure 1:**
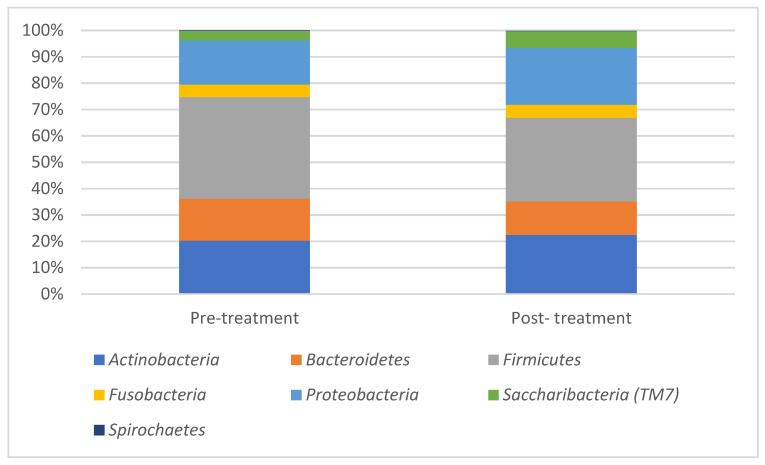
Presents mean results for microbiome pre- and post-treatment at phylum level.

**Figure 2:**
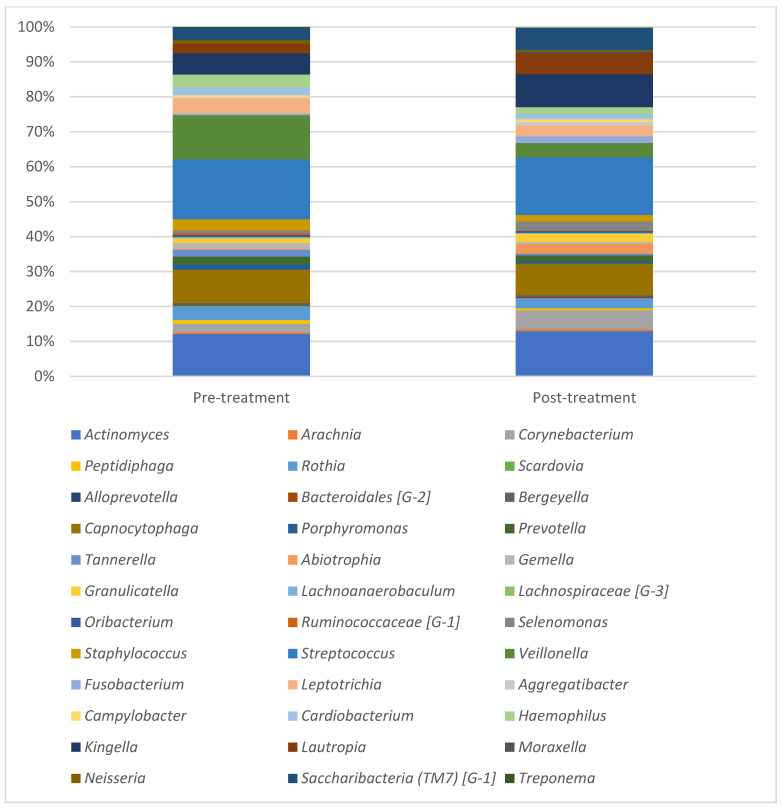
Presents mean results for microbiome pre- and post-treatment at genera level.

Bacterial distribution at phyla and genera levels for pre- and post-treatment samples are presented in Figure 1 and Figure 2. At the phyla level, pre-treatment samples showed a higher prevalence of *Firmicutes* (38%) and *Bacteroidetes* (15%) as compared with the post-treatment samples (31 and 12%, respectively). At genera level, the genera *Veillonella* that showed an abundance of 12% in the pre-treatment samples was reduced to 4% in the post-treatment samples.

**Figure 3:**
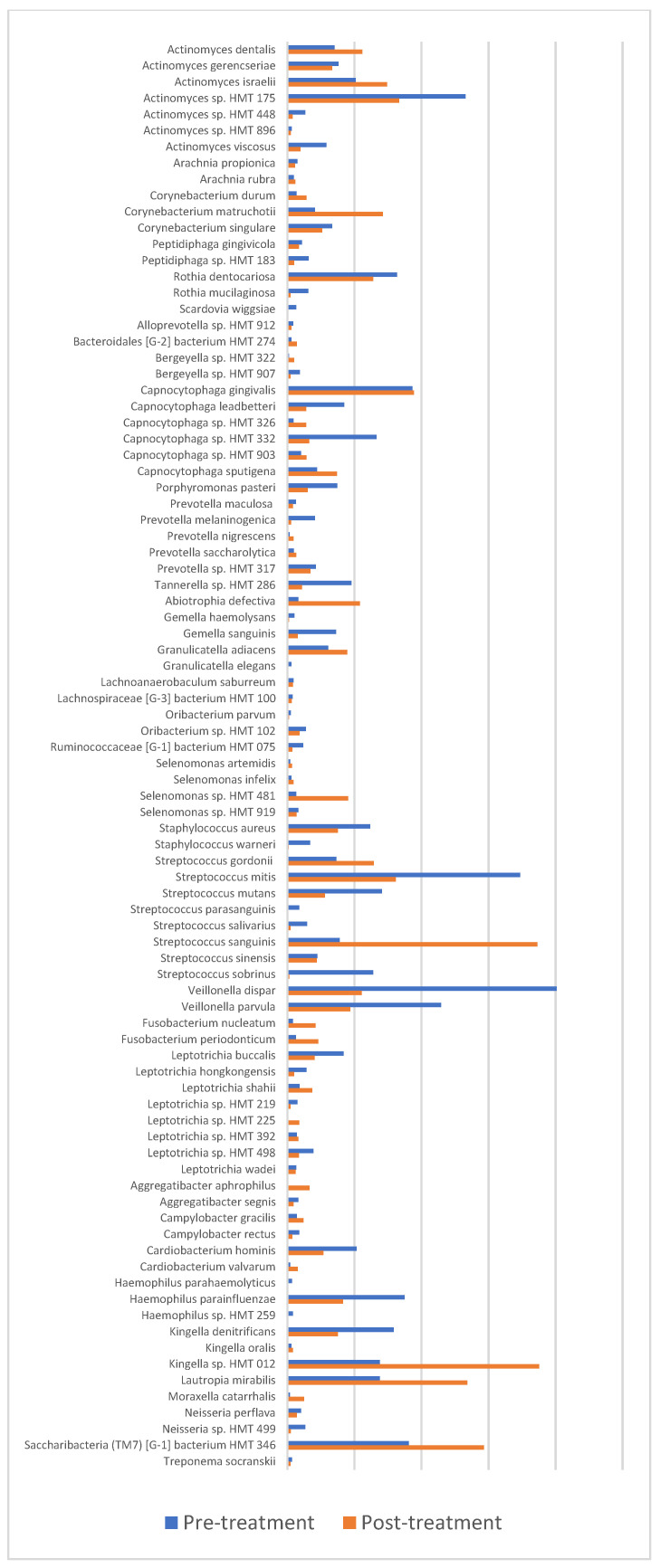
Presents microbiome pre- and post-treatment at Species level.

Results of the bacterial species analysis are presented in Figure 3. These results demonstrate a reduction in the abundance of cariogenic bacteria: *Streptococcus mutans*, *Streptococcus sobrinus*, *Rothia dentocariosa* and *Actinomyces viscosus,* ranging from 22% to over 90% reduction in the post-treatment follow-up samples as compared with pre-treatment samples. However, these reductions were not statistically significant and only one cariogenic bacterium (i.e., *Scardovia wiggisiae*) was significantly (*p* < 0.01) reduced to below detection levels. *Veillonella* spp. were also reduced by 60–70% in the follow up samples (*p* = 0.02). Interestingly, both baseline and follow up samples showed abundant population of obligate anaerobes implicated as periodontal pathogenic genera such as *Porphyromonas*, *Prevotella*, *Tanerella* and *Treponema* all indicators of mature pathogenic biofilm.

## 4. Discussion

Dental caries is the result of a floral shift in the dental plaque, from a balanced eubaiotic bacterial population to a dysbiotic cariogenic microbial population developed and supported by frequent exposures to sucrose and other fermentable dietary carbohydrates. This shift corresponds with an imbalance between demineralization and remineralization processes, leading to net mineral loss in the dental hard tissues resulting in the formation of the carious lesion [24].

Dental caries is a multifactorial disease resulting from the combined effect of various risk factors that take part in its progression. These may include risk factors such as cariogenic bacteria, insufficient salivary flow, bad oral hygiene, a high sugar diet and environmental factors including low socioeconomic status, premature birth or low birth weight [25,26,27].

ECC is an aggressive form of dental caries that affects the primary dentition of young children, typically involving the anterior tooth surfaces and can affect maxillary or mandibular primary molars. Initially, white spot lesions are formed in upper primary incisors along the margin of the gingiva. If allowed to progress, caries lesion can develop from a cavitated state to a complete destruction of the tooth crown. In severe cases, the carious process results in complete destruction of the upper teeth and spreads to the lower molars. ECC may lead to pain, infections, destruction of the dentition, reduction in life quality of children (difficulties in speaking, sleeping, eating, and delayed physical growth) and school and social disruption for children and their caregivers when left untreated [6].

Dental treatment of caries in the primary dentition traditionally involves restorations, pulp treatment and extraction of non-restorable teeth. Because of young children’s inability to tolerate comprehensive treatments in the dental chair, many affected children require treatment under GA [14].

This pilot study was designed to establish the effect of a full-mouth comprehensive dental rehabilitation under GA on the microbiota of children with S-ECC with emphasis on the effect of this treatment on the prevalence of cariogenic bacteria. There is ample evidence associating between *Streptococcus mutans* and dental caries. It is commonly known that the cariogenicity of *Streptococcus mutans* is due to its superior acidogenic and aciduric potential. The ability of mutans-group streptococci to produce water-insoluble glucan and glucan binding proteins helps their persistence in the dental biofilm [3]. However, recent studies conducted using pyrosequencing on childhood caries reported that the microbiota identified in infected tissues showed elevated abundance of several bacterial genera including *Actinomyces*, *Lactobacillus*, *Megasphaera*, *Olsenella*, *Scardovia*, *Shuttleworthia*, *Cryptobacterium*, and *Streptococcus* and tended to be stage-specific during the development of carious lesion [28,29].

Our results demonstrated a slight reduction in the abundance of cariogenic bacteria (*Streptococcus mutans*, *Streptococcus sobrinus*, *Rothia dentocariosa*, *Scardovia wiggisiae* and *Actinomyces viscosus*) in the dental biofilm samples of the examined children, 3 months after a full-mouth dental treatment. However, these reductions were not statistically significant, and for the most part these children maintained a cariogenic flora. These findings are in agreement with the results of Tanner et al. who confirmed a reduction in several species in addition to *Streptococcus mutans* in the microbiota of children without new caries lesions after treatment [30]. However, they also suggested that children with S-ECC, who had different species in their pre-treatment microbiota, maintained the same composition of microbiome following treatment, which was responsible for caries progression in that group in the first place.

Lactobacilli form a group of organisms often isolated from advancing carious lesions [3]. In the present study, we did not find Lactobacilli in the biofilm of the children pre- and post-treatment. Indeed, Lactobacilli were considered as promotors of the carious process, but the current understanding is that *mutans streptococci* group play a crucial role in the early stages of dental caries progress followed by colonization of Lactobacilli in deeper lesions [31,32]. It is possible that our method of sampling (i.e., plaque samples) is not enough to provide a full picture and infected dentine should be sampled as well.

Interestingly, we found a reduction of 60–70% in *Veillonella* spp. which, might suggest a reduction in lactic acid production in the biofilm that is an important energy source for these bacteria. It has been previously reported that plaque pH increased half a year after initial examination but returned to its original status a year after the initial examination [3].

Both pre- and post-treatment samples demonstrated an abundance of obligate anaerobes and spirochetes (*Porphyromonas*, *Prevotella*, *Tanerella* and *Treponema*). The latter are characteristic of mature pathogenic biofilm and are associated with the lack of oral hygiene [33]. This is in consistency with the high plaque index scores (data not shown) seen in the five children participating in the study and may suggest that the oral hygiene practice in these children was lacking and did not improve following treatment.

The aggressive dental approach of ECC under GA (extractions, pulp therapy and *stainless* steel crowns) contributes to a significant change quality of life, i.e., in pain complaint, eating preferences, quantity of food eaten and sleep habits before vs. after dental treatment among young children. Furthermore, GA usually allows treatment to be accomplished under optimal conditions; thus, the expectations for an ideal outcome, especially in restorative treatments, are high. However, many of the children with ECC that are treated under GA display high decline rates ranging from 51% in the high attendance patients to 68% in patients with lower attendance rates [34] other clinicians reported a relapse rate as high as 79.7% 12 months after dental treatment under GA [20]. These might be explained by a lack in follow up care and persistence of cariogenic habits post-GA dental treatment [35,36].

Prevention for ECC should begin during infancy. Physicians, nurses and other health caretakers may have more opportunities to teach the parent/caregiver than dental professionals due to the frequency of contact with the family in the child’s first year of life. A more comprehensive approach to the treatment of ECC including parental involvement might aid and promote preventive measures while encouraging the identification and reduction of individual caries risk factors. Since children who experience ECC are at greater risk for caries development, preventive measures (e.g., dietary counseling and reinforcement of tooth-brushing with fluoridated toothpaste), more frequent professional visits with applications of high concentrated topical fluoride and restorative care are necessary [6].

In conclusion, within the limitations of a pilot study conducted on a small number of cases, the results of the present study suggest that despite a mild reduction in the abundance of cariogenic bacteria following full-mouth dental rehabilitation under GA for children with S-ECC, the overall microbial dysbiotic state persisted, implying that additional measures are needed in order to prevent relapse in children suffering from S-ECC.

## Figures and Tables

**Table 1:** Patients’ information and caries experience index.

Subject No	Gender	Age (Months)	ASA ^1^	dmfT before GA				dmfT after GA			
				d	m	f	Total dmfT ^2^	d	m	f	Total dmfT ^2^
1	Male	61	1	11	0	0	11	0	2	9	11
2	Female	37	1	13	0	0	13	0	4	9	13
3	Male	50	1	13	0	0	13	0	1	12	13
4	Male	38	1	18	0	0	18	0	2	16	18
5	Male	41	1	12	0	0	12	0	1	11	12

^1^ ASA—American Society of Anesthesiology [23]. ^2^ d—decayed, m—missing, f—filled, T—tooth.

## Data Availability

The data presented in this study are available on request from the corresponding author. The data are not publicly available due to patients’ privacy.
